# The T2‐FLAIR mismatch sign as an imaging biomarker for oligodendrogliomas in dogs

**DOI:** 10.1111/jvim.16749

**Published:** 2023-05-29

**Authors:** Josefa Garcia‐Mora, Rell L. Parker, Thomas Cecere, John L. Robertson, John H. Rossmeisl

**Affiliations:** ^1^ Department of Small Animal Clinical Sciences and Animal Cancer Care and Research Center Virginia‐Maryland College of Veterinary Medicine, Virginia Tech Blacksburg Virginia USA; ^2^ Veterinary and Comparative Neuro‐Oncology Laboratory, Virginia‐Maryland College of Veterinary Medicine, Virginia Tech Blacksburg Virginia USA; ^3^ Department of Biomedical Sciences & Pathobiology Virginia‐Maryland College of Veterinary Medicine, Virginia Tech Blacksburg Virginia USA; ^4^ School of Biomedical Engineering and Sciences, Virginia Tech‐Wake Forest University Blacksburg Virginia USA; ^5^ Comprehensive Cancer Center and Brain Tumor Center of Excellence, Wake Forest School of Medicine Winston‐Salem North Carolina USA

**Keywords:** brain tumor, CNS disorders, magnetic resonance imaging, neuroimaging, neurology, oncology‐diagnosis, radiology and diagnostic imaging

## Abstract

**Background:**

In humans, the T2‐weighted (T2W)—fluid‐attenuated inversion recovery (FLAIR) mismatch sign (T2FMM) is a specific imaging biomarker for the isocitrate dehydrogenase 1 (IDH1)‐mutated, 1p/19q non‐codeleted low‐grade astrocytomas (LGA). The T2FMM is characterized by a homogeneous hyperintense T2W signal and a hypointense signal with a hyperintense peripheral rim on FLAIR sequences. In gliomas in dogs, the T2FMM has not been described.

**Hypotheses/Objectives:**

In dogs with focal intra‐axial brain lesions, T2FMM will discriminate gliomas from other lesions. The T2FMM will be associated with the LGA phenotype and presence of microcysts on histopathology. Interobserver agreement for T2FMM magnetic resonance imaging (MRI) features will be high.

**Animals:**

One hundred eighty‐six dogs with histopathologically diagnosed focal intra‐axial lesions on brain MRI including oligodendrogliomas (n = 90), astrocytomas (n = 47), undefined gliomas (n = 9), cerebrovascular accidents (n = 33), and inflammatory lesions (n = 7).

**Methods:**

Two blinded raters evaluated the 186 MRI studies and identified cases with the T2FMM. Histopathologic and immunohistochemical slides of T2FMM cases were evaluated for morphologic features and IDH1‐mutations and compared to cases without the T2FMM. Gene expression analyses were performed on a subset of oligodendrogliomas (n = 10) with and without T2FMM.

**Results:**

The T2FMM was identified in 14/186 (8%) of MRI studies, and all dogs with T2FMM had oligodendrogliomas (n = 12 low‐grade [LGO], n = 2 high‐grade [HGO]; *P* < .001). Microcystic change was significantly associated with the T2FMM (*P* < .00001). In oligodendrogliomas with T2FMM, IDH1‐mutations or specific differentially expressed genes were not identified.

**Conclusion and Clinical Importance:**

The T2FMM can be readily identified on routinely obtained MRI sequences. It is a specific biomarker for oligodendroglioma in dogs, and was significantly associated with non‐enhancing LGO.

**Abbreviations**
CVA: cerebrovascular accident
DEGs: differentially expressed genes
HGO: high‐grade oligodendroglioma
LGA: low‐grade astrocytoma
LGO: low‐grade oligodendroglioma
MUE: meningoencephalitis of unknown etiology
T2FMM: T2‐FLAIR mismatch sign

## 1 INTRODUCTION

In dogs, gliomas account for 35% of primary brain tumors, and feature remarkable neuroimaging, clinical, and phenotypic similarities to gliomas in humans, making them an excellent model for studies in humans.^1^, ^2^, ^3^, ^4^ Characterizing glioma genetic drivers in dogs and identifying molecular determinants common to humans and dogs only recently has been investigated.^4^

Glioma classification in humans is based on 2021 World Health Organization criteria, including genotypic, molecular and phenotypic tumor features, describing entities in which the presence of isocitrate dehydrogenase 1/2 (IDH1/2) mutations and 1p/19q codeletion status are considered important predictors of positive prognosis and longer overall survival than are found in patients carrying the wild‐type IDH1/2 gene.^5^, ^6^, ^7^ Genomic features of gliomas in humans also have been associated with certain magnetic resonance imaging (MRI) biomarkers such as the T2‐weighted (T2W)—fluid‐attenuated inversion recovery (FLAIR) mismatch (T2FMM) sign, which has high to perfect specificity and moderate to low sensitivity to predict IDH1‐mutant, 1p/19q non‐codeleted low‐grade astrocytomas (LGA).^8^, ^9^, ^10^, ^11^, ^12^, ^13^ The T2FMM is characterized by a homogeneous hyperintense signal on T2W and a hypointense signal with a hyperintense peripheral rim on FLAIR.^8^, ^13^

Although histopathology is the gold standard to diagnose gliomas, antemortem diagnosis by biopsy rarely is performed in veterinary medicine because of practical limitations, such as availability of centers with experience performing brain biopsy or surgery in dogs with gliomas.^1^, ^12^, ^13^, ^14^, ^15^, ^16^ Hence, the antemortem diagnosis of gliomas is often presumptive based on MRI features.^1^, ^2^, ^16^, ^17^ A recent classification scheme for gliomas in dogs recognizes tumor types as oligodendrogliomas, astrocytomas, or undefined gliomas, and classifies them as low‐grade or high‐grade according to their morphologic features.^3^ Although the qualitative MRI features of gliomas in dogs have been widely studied, no reliable imaging features distinguish among tumor types or grades, but tumor contrast enhancement occurs more frequently in high‐grade than low‐grade gliomas.^1^, ^2^, ^18^, ^19^ A lack of biological specificity in MRI signals has hindered identification of MRI biomarkers for glioma in dogs, which share imaging characteristics with other intracranial diseases such as abscesses, granulomas, meningoencephalitis of unknown etiology, and cerebrovascular accidents (CVA).^1^, ^2^, ^20^, ^21^ The T2FMM and its possible association with tumor phenotype have not been investigated previously in dogs.

The purpose of our study was to evaluate the presence of T2FMM in dogs with clinical and MRI evidence of focal intra‐axial brain diseases that can mimic gliomas. We hypothesized that the presence of the T2FMM would: (a) discriminate gliomas from other intra‐axial brain lesions; (b) be predictive of LGA phenotype; and (c) be associated with microcysts on histopathology of the mismatched tumor regions. Additionally, we investigated whether the T2FMM sign was associated with a particular genotype in a subset of dogs with gliomas.

## 2 MATERIALS AND METHODS

### 2.1 Study design and animals

Ours was a retrospective study of dogs with histologically confirmed, focal intra‐axial forebrain lesions associated with clinical signs of intracranial disease that were referred to our neurology practice between 2004 and 2022 for biopsy of their brain lesions or for treatment of presumed intracranial glioma.^16^ An institutional clinical trials database was searched for cases that met the following inclusion criteria: clinical signs of intracranial disease, presence of a focal intra‐axial forebrain lesion on brain MRI examination, and histopathologic confirmation of the lesion by stereotactic brain biopsy, surgical brain biopsy, or necropsy.^16^, ^21^ Because dogs were referred from multiple institutions, MRI scans were performed on 0.2T, 1.0T, 1.5T or 3T units, and MRI sequences obtained were not standardized. At a minimum, qualitative evaluations of conventional MRI images included T1‐weighted (T1W) and T1W contrast‐enhanced images in at least 2 planes, T2‐weighted (T2W) images in 2 planes, and FLAIR sequences. Proton density (PD), T2W gradient echo images (T2*GRE), and diffusion weighted imaging with anisotropic diffusion coefficient (DWI/ADC) sequences were reviewed when available. Images were reviewed independently by 2 raters including a board‐certified neurologist with more than 25 years of experience (JHR) and a veterinarian with 2 years of experience (JGM). The MR images were evaluated for the presence or absence of the core features of the T2FMM sign including a complete or nearly complete homogeneously hyperintense lesion signal on T2W sequences, and a complete or nearly complete relatively hypointense central signal except for a hyperintense peripheral rim on FLAIR.^8^, ^13^ To mitigate possible variability associated with qualitative reviewer interpretations of “complete” or “near‐complete” FLAIR signal suppression, we also required that ≥80% of the high T2W signal volume was FLAIR null (*F*_null_) to be considered a T2FMM positive case, as calculated using the equation:

$$F_{\text{null}}\text{Ratio}=\frac{F_{\text{null}}\;V}{\mathrm{TTV}},$$

where the *F*_null_ *V* is the lesion volume that suppressed on FLAIR sequences and TTV is the total T2W lesion volume.^22^ The *F*_null_ *V* and TTV were determined using commercial image analysis software (Osirix MD, v11.0.4, Pixmeo, Switzerland) from transverse sequences using manually defined regions of interest (ROIs) from contiguous slices representing the hypointense (null) portion of the lesion on FLAIR sequences and the homogenously hyperintense portion of the lesion on T2W images, respectively.^22^ The *F*_null_ *V* and TTV then were calculated using the software's ROI volume function. Additionally, T1W signal intensity (hypo‐, iso‐, or hyperintense) and presence (present or absent) of lesion contrast enhancement were assessed.

### 2.2 Histopathologic and immunohistochemical analyses

Histopathologic diagnoses for all lesions were recoded from the surgical pathology or necropsy reports in medical records. A consensus review of all gliomas was performed by 2 veterinary pathologists using hematoxylin and eosin, and when requested, glial fibrillary acidic protein or oligodendrocyte transcription factor 2 (OLIG‐2)‐stained sections to type and grade gliomas and assess and record phenotypic features of gliomas in dogs.^3^, ^16^ Additionally, immunohistochemistry for the IDH1 Arg132His mutation was performed on all cases with T2FMM and 15 other randomly selected oligodendrogliomas without T2FMM (random number generator, Prism 9.5.0, GraphPad LLC; low‐grade oligodendroglioma [LGO = 8] and high‐grade oligodendrogliomas [HGO = 7]), as described previously.^23^ Briefly, 4 μm sections from each tumor block were cut using a microtome, and antigen retrieval was performed using a citrate buffer at pH 6.0 for 60 minutes. Slides were dried at 80°C for 15 minutes, incubated with IDH1 R132H antibody (1:150 dilution, mouse monoclonal OTI3G9 [Origene, Rockville, Maryland, USA]), for 30 minutes at 37°C. For chromogenic detection, a universal 3,3′Diaminobenzidine (DAB) detection kit (Ventana Medical Systems, Tucson, Arizona, USA) was used. An IDH1‐mutant genotyped grade IV astrocytoma from a human was used as a positive control, and the primary antibody was omitted from negative controls.

### 2.3 RNA isolation and gene expression analyses

Total RNA was isolated from 20 mg frozen oligodendroglioma samples (n = 5 with T2FMM [4 LGO and 1 HGO] and n = 5 without the T2FMM [2 LGO and 3 HGO]) using an AllPrepDNA/RNA Mini Kit (Qiagen, Valencia, California, USA), according to the manufacturer's instructions. Additional DNase treatment was performed on‐column for RNA purification. We also extracted RNA from formalin fixed, paraffin embedded (FFPE) tissue scrolls from an additional 9 canine oligodendrogliomas (n = 4 with T2FMM and n = 5 without T2FMM) using the AllPrep DNA/RNA FFPE Kit (Qiagen). The RNA sample concentration was determined using a NanoDrop ND‐1000 spectrophotometer (NanoDrop Technologies, Wilmington, Delaware, USA) and quality was measured using a 2100 Bioanalyzer (Agilent, Santa Clara, California, USA). Microarray gene expression profiling was performed with 4 μg of RNA labeled using the Affymetrix labeling protocol (Applied Biosystems, Waltham, Massachusetts, USA). Biotinylated cRNA was generated, amplified, and labeled following the protocol described in GeneChip Eukaryotic Small Sample Target Labeling Assay (v2; www.affymetrix.com). The cRNA samples then were hybridized to Affymetrix GeneChip Canine Genome 2.0 microarrays (Applied Biosystems) following the manufacturer's protocol. A high‐density GeneChip Scanner 3000 was used to obtain raw data. Quality control was performed using Affymetrix's recommended measures and evaluations enabled by the software packages made4, affy and affyPLM. Ultimately, 8/9 RNA extractions from FFPE samples did not pass quality control, and thus only the RNA samples derived from frozen tumors (n = 10) were analyzed. Comparisons of gene expression between oligodendrogliomas from dogs with and without the T2FMM sign were performed using the edge R package. Differentially expressed genes (DEG) were identified using volcano plot filters, with a false discovery rate screening threshold of <0.01 and fold‐change (FC) ≥2.

### 2.4 Statistical analysis

Fisher exact tests were used to compare the proportions of binary MRI features present, breed, sex, tumor anatomic location and clinical presentation distributions in dogs with oligodendrogliomas with and without the T2FMM. Interobserver agreement for qualitative MRI features defining T2FMM was evaluated by calculating intraclass correlation coefficients. Two sample *z*‐tests were performed to evaluate association between histological phenotypic features and the T2FMM sign. Gene expression between oligodendrogliomas in dogs with and without the T2FMM sign was compared using a paired *t* test, and *P* values <.05 were considered significant.

## 3 RESULTS

### 3.1 Diagnoses in dogs with focal intra‐axial brain lesions

Data from 186 dogs were included in the study. Histopathologic diagnoses included 90 oligodendrogliomas (36 LGO; 54 HGO), 47 astrocytomas (17 LGA; 30 high‐grade astrocytomas [HGA], 9 undefined gliomas), 33 CVA, 3 abscesses, and 4 granulomas.

### 3.2 T2FMM MRI assessment, interobserver agreement, and clinical findings

**FIGURE 1:**
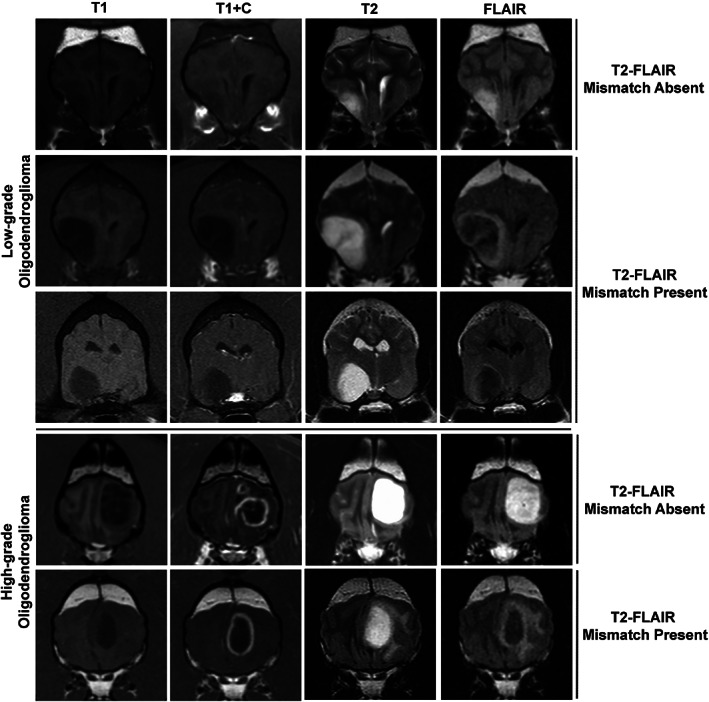
Magnetic resonance imaging features of canine low‐grade (top three rows) and high‐grade (bottom two rows) oligodendrogliomas in which the T2FMM sign was present and absent. The T2FMM sign is characterized by a homogeneous hyperintense T2W signal and a hypointense signal with a hyperintense peripheral rim on FLAIR sequences. T2FMM, T2‐fluid‐attenuated inversion recovery mismatch sign.

The T2FMM was identified in 8% (14/186) of brain MRI studies in dogs with focal intra‐axial lesions, and was exclusively identified in oligodendrogliomas (Table 1). The T2FMM was identified in 16% (14/90) of all oligodendrogliomas in dogs in the dataset. For the detection of oligodendroglioma, the T2FMM specificity was 100% and the sensitivity 16%. The presence of the T2FMM sign was significantly associated with low‐grade, non‐contrast enhancing oligodendrogliomas that did not contain T2*GRE signal voids (Table 1; Figure 1). Because all oligodendrogliomas in the dataset were hyperintense on PD images and none of the oligodendrogliomas featured restricted diffusion, PD and DWI/ADC imaging features were not included in statistical analyses. Interobserver agreement for detecting qualitative features of T2FMM was good to excellent (Table 2).

**TABLE 1:** Prevalence, diagnoses, and MRI features of the T2‐FLAIR mismatch sign in 186 dogs with focal, intra‐axial brain lesions.

	T2‐FLAIR mismatch sign present	T2‐FLAIR mismatch sign absent	*P*‐value
	Frequency (%)	Frequency (%)	
All diagnoses, n = 186	14 (8%)	172 (92%)	
Astrocytoma, n = 47	0 (0%)	47 (100%)	<.0001*
Undefined glioma, n = 9	0 (0%)	9 (100%)	<.0001*
Abscess/Granuloma, n = 7	0 (0%)	7 (100%)	<.0001*
Infarction, n = 33	0 (0%)	33 (100%)	<.0001*
Oligodendroglioma grade, n = 90	14 (16%)	76 (84%)	Referent
Low‐grade oligodendroglioma, n = 36	12 (33%)	24 (67%)	.001*
High‐grade oligodendroglioma, n = 54	2 (4%)	52 (96%)	
Oligodendroglioma T2*GRE signal voids, n = 90			
Present, n = 23	0 (0%)	23 (100%)	<.0001*
Absent, n = 67	14 (21%)	53 (79%)	
Oligodendroglioma contrast enhancement, n = 90			
Non‐enhancing, n = 19	11 (58%)	8 (42%)	.002*
Enhancing, n = 71	3 (4%)	68 (96%)	
Oligodendroglioma T1 signal, n = 90			
Hypointense, n = 84	13 (15%)	71 (85%)	.42
Isointense, n = 6	1 (16%)	5 (83%)	

Abbreviations: GRE, gradient echo image; MRI, magnetic resonance imaging; T2‐FLAIR, T2‐fluid‐attenuated inversion recovery.

* Statistically significant (*P* < .05), Fisher exact test.

**TABLE 2:** Interobserver intraclass correlations (ICC) of MRI features defining the T2‐FLAIR mismatch sign.

Lesion feature	ICC
T2W homogeneous hyperintensity	.96
Hyperintense FLAIR peripheral rim	1.00
Central hypointense FLAIR signal (>80% FLAIR volume null)	.81

Abbreviations: MRI, magnetic resonance imaging; T2‐FLAIR, T2‐fluid‐attenuated inversion recovery.

Among the 14/90 dogs with oligodendrogliomas displaying the T2FMM, there were 3 Boxers (21%), 3 mixed breed dogs (21%), 3 French bulldogs (21%), 2 Boston terriers (14%), 2 English bulldogs (14%), and 1 Cane Corso (7%), and 8/14 (57%) dogs were castrated males and 6/14 dogs (43%) spayed females. The median age of dogs with T2FMM was 7.4 years (range, 5‐11 years) and the median body weight was 19.6 kg (range, 9‐51 kg). Eleven dogs (11/14; 79%) with T2FMM presented with seizures and interictal signs of forebrain disease, and 3 dogs (3/14; 21%) presented with clinical signs of focal forebrain disease only (circling, hemi‐inattention, thalamocortical visual deficits). Five T2FMM tumors (5/14; 36%) were located in the frontal lobe, 3/14 (21%) in the temporal‐piriform lobes, 3/14 (21%) in the fronto‐olfactory lobes, 2/14 (14%) in the parieto‐temporal lobes, and 1/14 (7%) in the temporal‐occipital lobes.

Of the 76/90 dogs with oligodendrogliomas without the T2FMM there were 19 Boxers (25%), 13 mixed breeds (17%), 10 Boston terriers (13%), 8 American Staffordshire terriers (11%), 8 French bulldogs (11%), 5 English bulldogs (6%), 3 Labrador retrievers (4%), and 10 other pure breeds represented by 1 case each. The median age of dogs without T2FMM was 8.1 years (range, 4‐13 years) and the median body weight was 22.8 kg (range, 4‐60 kg). Among the oligodendrogliomas without the T2FMM sign, 58/76 (76%) presented with seizures with (39/58 [67%]) or without (19/58 [33%]) interictal signs of forebrain disease, and 18/76 (24%) presented with clinical signs of forebrain diseases without a history of seizures. Twenty of 76 tumors (26%) were located in the frontal lobe, 17/76 (22%) were located in the frontal‐olfactory lobes, 15/76 (20%) were located in the parieto‐temporal lobes, 13/76 (17%) were in the temporal‐piriform lobes, 7/76 (9%) tumors involved ≥3 lobes, and 4/76 (5%) were found in the temporal‐occipital lobes. No significant differences were observed in age (*P* = .59), body weight (*P* = .48), breed (*P* = .61) or sex (*P* = .77) distributions, clinical signs (*P* = .76), or tumor anatomic locations (*P* = .63) between dogs with and without the T2FMM.

### 3.3 T2FMM histopathologic and immunohistochemical analyses

Significant correlations between the presence of microcysts and myxoid matrix lakes were found in oligodendrogliomas presenting with the T2FMM sign (Table 3). No other grade agnostic histopathological features of oligodendrogliomas were significantly associated with the T2FMM sign.

**TABLE 3:** Grade agnostic histological features of canine oligodendrogliomas with and without the T2‐FLAIR mismatch sign.

Histologic feature by tumor grade	Frequency T2‐FLAIR mismatch present (%)	Frequency T2‐FLAIR mismatch absent (%)	*P*‐value
Low‐grade, total n = 36	n = 12	n = 24	
Intrafascicular rowing	3/12 (25%)	10/24 (42%)	.32
Microcalcifications	4/12 (33%)	9/24 (38%)	.80
Microcystic change	12/12 (100%)	7/24 (29%)	<.00001*
Myxoid matrix lakes	12/12 (100%)	4/24 (17%)	<.00001*
Pseudorosettes	0/12 (0%)	1/24 (4%)	.47
Wickerwork vasculature	1/12 (8%)	6/24 (25%)	.23
High‐grade, total n = 54	n = 2	n = 52	
Intrafascicular rowing	0/2 (0%)	19/52 (37%)	.29
Microcalcifications	1/2 (50%)	22/52 (42%)	.83
Microcystic change	2/2 (100%)	16/52 (46%)	.04*
Myxoid matrix lakes	2/2 (100%)	13/52 (25%)	.02*
Pseudorosettes	1/2 (50%)	7/52 (15%)	.15
Wickerwork vasculature	0/2 (0%)	11/52 (21%)	.46

* Statistically significant (*P* < .05), two sample *z*‐test.

### 3.4 Immunohistochemical and molecular analysis

**FIGURE 2:**
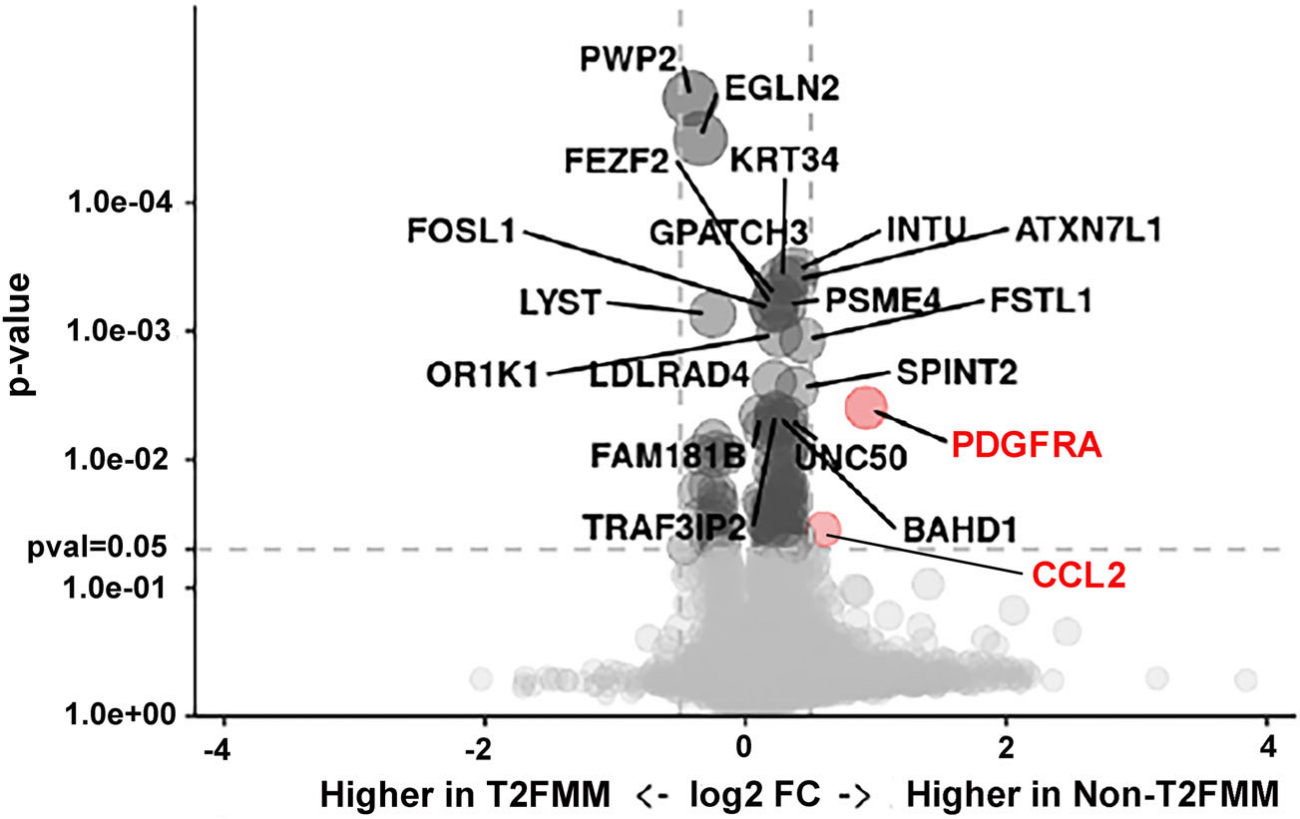
Volcano plot showing cutoffs of false discover rate‐adjusted *P*‐values and fold change for all genes (gray circles) and differentially expressed (red circles) between canine oligodendrogliomas with and without the T2FMM. T2FMM, T2‐fluid‐attenuated inversion recovery mismatch sign.

Immunoreactivity to the IDH1‐mutation was not observed in any the oligodendrogliomas with (n = 0/14) or without (n = 0/15) the T2FMM sign. Using volcano plot analyses, 2 DEGs, *PDGFRA* and *CCL2* were identified between dogs with and without the T2FMM (Figure 2), both of which were upregulated in dogs with oligodendrogliomas without the T2FMM.

## 4 DISCUSSION

Although our initial hypothesis associating the T2FMM sign with the LGA phenotype in dogs was rejected, our results indicate that the T2FMM is a highly specific indicator of oligodendrogliomas in dogs, particularly non‐enhancing LGO phenotypes. Evaluation of the T2FMM sign is simple and it can be easily identified by evaluators with a range of experience using routinely obtained conventional MRI sequences. We evaluated the performance of the T2FMM sign as a biomarker in a large population of dogs with different intra‐axial brain diseases, in contrast with prior studies that considered selected groups of dogs with only gliomas or oligodendrogliomas.^2^, ^18^, ^19^ Therefore, our results are more clinically relevant because they are based on a more representative sample of dogs with intracranial diseases that can mimic gliomas seen in clinical practice.

Previous studies have indicated that no MRI features reliably predict glioma phenotypes or grades in populations that included dogs with astrocytomas and oligodendrogliomas, although the presence of contrast enhancement has been associated with high‐grade tumors.^1^, ^2^, ^15^, ^18^ Although MRI features compatible with the T2FMM sign previously were included in a study of oligodendrogliomas in dogs, ours is the first study that specifically identified a significant relationship between an MRI biomarker and the oligodendroglial phenotype in dogs.^18^ In adult humans, the T2FMM sign is a highly accurate biomarker for IDH1‐mutated, 1p/19q non‐codeleted astrocytomas, with specificity and sensitivity as high as 100% and 31%, respectively.^8^, ^9^, ^10^, ^11^, ^12^ We found a similar high specificity and low sensitivity for the T2FMM to detect oligodendrogliomas in dogs. It is important to note that the T2FMM sign has been reported in other tumor types in humans, such as IDH1‐mutated, 1p/19q co‐deleted oligodendrogliomas and IDH1 wild‐type glioblastomas.^11^, ^13^ Although dogs remain a valuable animal model for comparative studies of glioma, our study adds additional evidence of important differences in glioma biology between dogs and humans, because we did not identify associations between T2FMM and astrocytomas in dogs or the presence of IDH1 mutations.

One possible reason for our discordant T2FMM findings relative to humans may be that gliomas in dogs have been shown to be more closely related to gliomas in children at the genetic and epigenetic levels, and considerable pathophysiological differences exist between glioma presentations in human adults and children.^4^ We did not identify an association of the T2FMM with IDH1 mutations in dogs, but this finding was not unexpected given the rarity in which IDH1 mutations have been identified in dogs with gliomas.^1^, ^4^ Overexpression of *PDGFRA* has been shown to be a common driver of tumorigenesis in humans and dogs with gliomas, and to be present more frequently in high‐grade gliomas.^4^, ^23^, ^24^, ^25^, ^26^, ^27^, ^28^ In addition, *CCL2* is a known progression‐inducing factor in humans with gliomas and has been reported to be significantly upregulated in HGA.^23^ In our study, no specific genotype was associated with oligodendrogliomas exhibiting T2FMM, but *PDGFRA* and *CCL2* were significantly upregulated among oligodendrogliomas without the sign, which likely reflects sampling bias of inclusion of more HGO in the group without the T2FMM sign. We also did not assess for 1p/19q codeletions, because these have not yet been identified in dogs with gliomas.^4^ The small sample sizes included in the molecular analyses could have precluded us from identifying particular genotypes associated with the T2FMM.

Our results highlight that the primary value of the T2FMM in dogs is its specificity for oligodendroglial tumors, rather than its sensitivity. By using more rigid criteria for the T2FMM sign in dogs, we hoped to avoid difficulties that have been encountered in studies in humans that applied purely qualitative definitions of T2FMM to cases, which have subsequently resulted in reductions in the specificity of this biomarker.^13^ Although inter‐rater agreements for T2FMM criteria were good to excellent in our study, the primary discordance between reviewers arose from analysis of the FLAIR null tumor burden in cases imaged on low‐field magnets. Magnetic field strength also can influence interpretation of T2FMM, because higher magnetic field strengths will result in more FLAIR signal attenuation, making the FLAIR null signal more obvious and thus F_null_ volumetric ROI easier to contour.^29^ Conversely, stronger magnetic fields also can influence interpretation of the T2W lesion signal, with glioma T2W signal being less homogenous on 3T than on 1.5T scanners.^13^

False positive cases of the T2FMM sign have been reported in children with gliomas, including pilomyxoid astrocytoma and K27M mutant midline gliomas, as well as non‐neoplastic diseases of the brain.^30^, ^31^, ^32^ In these tumors, T2FMM occurs because of the presence of microcystic changes in the tumor regions of mismatch, which is consistent with our findings because microcysts were present in all T2FMM cases reported here.^12^ The biological basis for the T2FMM appearance of microcysts is currently unknown, because microcysts also have been found in IDH1‐mutated, 1p/19q non‐codeleted astrocytomas and in oligodendrogliomas in dogs that do not present with the T2FMM sign.^12^ We also identified a significant association between the presence of myxoid matrix lakes and dogs with the T2FMM sign, a histologic feature that has not been correlated with this specific sign in humans. Myxoid tissue is variably present in oligodendrogliomas in dogs and in other non‐neural soft tissue neoplasms, and consists of a mucopolysaccharide matrix of sulfated and non‐sulfated glycosaminoglycans with high water content.^32^, ^33^ Myxoid lakes are also a rare microscopic finding in other rare types of neuroepithelial tumors, such as dysembryoplastic neuroepithelial tumor (DNET), a benign neuroglial tumor with a heterogeneous cellular composition, containing myxoid tissue.^34^, ^35^, ^36^ In humans with DNET an MRI feature very similar to the T2FMM sign is found, known as the “bright rim sign,” which is described as a complete or incomplete hyperintense well‐defined rim around the DNET on FLAIR sequence.^34^ Because DNETs also commonly have cystic structures and abundant myxoid matrix content, which make them appear hyperintense on T2W and hypointense on T1W, they can mimic the T2FMM sign.^34^

## 5 CONCLUSION

Although the presence of the T2FMM sign is uncommon in dogs with gliomas, it is an easily identifiable and specific imaging biomarker for oligodendrogliomas in dogs. Although we did not identify false positive cases of T2FMM in our study, in humans false positive cases occurring in other types of neuroepithelial tumors and non‐neoplastic diseases of the brain are reported.

## CONFLICT OF INTEREST DECLARATION

Authors declare no conflict of interest.

## OFF‐LABEL ANTIMICROBIAL DECLARATION

Authors declare no off‐label use of antimicrobials.

## INSTITUTIONAL ANIMAL CARE AND USE COMMITTEE (IACUC) OR OTHER APPROVAL DECLARATION

Approved by Virginia‐Maryland College of Veterinary Medicine IACUC, protocol numbers 17‐011 and 19‐240.

## HUMAN ETHICS APPROVAL DECLARATION

Authors declare human ethics approval was not needed for this study.

## References

[1] Miller AD , Miller CR , Rossmeisl JH . Canine primary intracranial cancer: A clinicopathologic and comparative review of glioma, meningioma, and choroid plexus tumors. Front Oncol. 2019;9:1151. 31788444 10.3389/fonc.2019.01151 PMC6856054

[2] Young BD , Levine JM , Porter BF , et al. Magnetic resonance imaging features of intracranial astrocytomas and oligodendrogliomas in dogs. Vet Radio Ultrasound. 2011;52:132‐141. 10.1111/j.1740-8261.2010.01758.x 21388463

[3] Koehler JW , Miller AD , Miller CR , et al. A revised diagnostic classification of canine glioma: Towards validation of the canine glioma patient as a naturally occurring preclinical model for human glioma. J Neuropathol Exp Neurol. 2018;77:1039‐1054. 30239918 10.1093/jnen/nly085 PMC6181180

[4] Amin SB , Anderson KJ , Boudreau CE , et al. Comparative molecular life history of spontaneous canine and human gliomas. Cancer Cell. 2020;37:243‐257. 32049048 10.1016/j.ccell.2020.01.004 PMC7132629

[5] Loius DN , Perry A , Wesseling P , et al. The 2021 WHO classification of tumors of the central nervous system: A summary. Neuro Oncol. 2021;23:1231‐1251. 34185076 10.1093/neuonc/noab106 PMC8328013

[6] Houillier C , Wang X , Kaloshi G , et al. IDH1 or IDH2 mutations predict longer survival and response to temozolomide in low‐grade gliomas. Neurology. 2010;75:1560‐1566. 20975057 10.1212/WNL.0b013e3181f96282

[7] Lin W , Qiu X , Sun P , et al. Association of IDH mutation and 1p19q co‐deletion with tumor immune microenvironment in lower‐grade glioma. Mol Ther Oncolytics. 2021;21:288‐302. 34141867 10.1016/j.omto.2021.04.010 PMC8167204

[8] Patel SH , Poisson LM , Brat DJ , et al. T2‐FLAIR mismatch, an imaging biomarker for IDH and 1p/19q status in lower‐grade gliomas: A TCGA/TCIA project. Clin Cancer Res. 2017;23(20):6078‐6085. 28751449 10.1158/1078-0432.CCR-17-0560

[9] Broen MPG , Smits M , Wijnenga MMJ , et al. The T2‐FLAIR mismatch sign as an imaging marker for non‐enhancing IDH‐mutant, 1p/19q‐intact lower‐grade glioma: a validation study. Neuro Oncol. 2018;20:1393‐1399. 29590424 10.1093/neuonc/noy048 PMC6120363

[10] Corell A , Ferreyra Vega S , Hoefling N , et al. The clinical significance of the T2‐FLAIR mismatch sign in grade II and III gliomas: a population‐based study. BMC Cancer. 2020;20:450. 32434559 10.1186/s12885-020-06951-w PMC7238512

[11] Do YA , Cho SJ , Choi BS , et al. Predictive accuracy of T2‐FLAIR mismatch sign for the IDH‐mutant, 1p/19q noncodeleted low‐grade glioma: An updated systematic review and meta‐analysis. Neurooncol Adv. 2022;4:vdac010. 35198981 10.1093/noajnl/vdac010 PMC8859831

[12] Deguchi S , Oishi T , Mitsuya K , et al. Clinicopathological analysis of T2‐FLAIR mismatch sign in lower‐grade gliomas. Sci Rep. 2020;10:10113. 32572107 10.1038/s41598-020-67244-7 PMC7308392

[13] Pinto C , Noronha C , Taipa R , Ramos C . T2‐FLAIR mismatch sign: a roadmap of pearls and pitfalls. Br J Radiol. 2022;95:20210825. 34618597 10.1259/bjr.20210825 PMC8722227

[14] Merickel JL , Pluhar GE , Rendahl A , O'Sullivan MG . Prognostic histopathologic features of canine glial tumors. Vet Pathol. 2021;58:945‐951. 34219560 10.1177/03009858211025795 PMC10923237

[15] José‐López R , Gutierrez‐Quintana R , de la Fuente C , et al. Clinical features, diagnosis, and survival analysis of dogs with glioma. J Vet Int Med. 2021;35:1902‐1917. 10.1111/jvim.16199 PMC8295679 34117807

[16] Kani Y , Cecere TE , Lahmers K , et al. Diagnostic accuracy of stereotactic brain biopsy for intracranial neoplasia in dogs: Comparison of biopsy, surgical resection, and necropsy specimens. J Vet Int Med. 2019;33:1384‐1391. 10.1111/jvim.15500 PMC6524398 30990928

[17] Debreuque M , De Fornel P , David I , et al. Definitive‐intent uniform megavoltage fractioned radiotherapy protocol for presumed canine intracranial gliomas: retrospective analysis of survival and prognostic factors in 38 cases (2013‐2019). BMC Vet Res. 2020;16:412. 33129320 10.1186/s12917-020-02614-x PMC7603708

[18] Amphimaque B , Durand A , Oevermann A , Vidondo B , Schweizer D . Grading of oligodendroglioma in dogs based on magnetic resonance imaging. J Vet Int Med. 2022;36:2104‐2112. 10.1111/jvim.16519 PMC9708455 36366870

[19] Stadler KL , Ruth JD , Pancotto TE , Werre SR , Rossmeisl JH . Computed tomography and magnetic resonance imaging are equivalent in mensuration and similarly inaccurate in grade and type predictability of canine intracranial gliomas. Front Vet Sci. 2017;4:157. 28993810 10.3389/fvets.2017.00157 PMC5622299

[20] Carloni A , Bernardini M , Mattei C , et al. Can MRI differentiate between ring‐enhancing gliomas and intra‐axial abscesses? Vet Radiol Ultrasound. 2022;63:563‐572. 35509117 10.1111/vru.13098

[21] Diangelo L , Cohen‐Gadol A , Heng HG , et al. Glioma mimics: magnetic resonance imaging characteristics of granulomas in dogs. Front Vet Sci. 2019;6:286. 31555671 10.3389/fvets.2019.00286 PMC6722480

[22] Mora JKG , Robertson JL , Hsu FC , et al. Comparison of linear and volumetric criteria for the determination of therapeutic response in dogs with intracranial gliomas. J Vet Int Med. 2022;36:1066‐1074. 10.1111/jvim.16406 PMC9151452 35274379

[23] Jahns H , McElroy MC . Bovine intracranial neoplasia: A retrospective case series. Vet Pathol. 2022;59:824‐835. 35638647 10.1177/03009858221100433 PMC9358308

[24] Connolly NP , Shetty AC , Stokum JA , et al. Cross‐species transcriptional analysis reveals conserved and host‐specific neoplastic processes in mammalian glioma. Sci Rep. 2018;8:1180. 29352201 10.1038/s41598-018-19451-6 PMC5775420

[25] Cancer Genome Atlas Research Network . Comprehensive genomic characterization defines human glioblastoma genes and core pathways. Nature. 2008;455:1061‐1068. 18772890 10.1038/nature07385 PMC2671642

[26] Paugh BS , Qu C , Jones C , et al. Integrated molecular genetic profiling of pediatric high‐grade gliomas reveals key differences with the adult disease. J Clin Oncol. 2010;28(18):3061‐3068. 20479398 10.1200/JCO.2009.26.7252 PMC2903336

[27] Koschmann C , Zamler D , MacKay A , et al. Characterizing and targeting PDGFRA alterations in pediatric high‐grade glioma. Oncotarget. 2016;7:65696‐65706. 27582545 10.18632/oncotarget.11602 PMC5323185

[28] Verhaak RG , Hoadley KA , Purdom E , et al. Cancer Genome Atlas Research Network. Integrated genomic analysis identifies clinically relevant subtypes of glioblastoma characterized by abnormalities in PDGFRA, IDH1, EGFR, and NF1. Cancer Cell. 2010;17:98‐110. 20129251 10.1016/j.ccr.2009.12.020 PMC2818769

[29] Hori M , Hagiwara A , Goto M , Wada A , Aoki S . Low‐field magnetic resonance imaging. Invest Radiol. 2021;56:659‐669. 10.1097/RLI.0000000000000810 PMC8505165 34292257

[30] Toedebusch R , Grodzki AC , Dickinson PJ , et al. Glioma‐associated microglia/macrophages augment tumorigenicity in canine astrocytoma, a naturally occurring model of human glioma. Neurooncol Adv. 2021;3(1):vdab062. 34131649 10.1093/noajnl/vdab062 PMC8193901

[31] Johnson DR , Kaufmann TJ , Patel SH , Chi AS , Snuderl M , Jain R . There is an exception to every rule‐T2‐FLAIR mismatch sign in gliomas. Neuroradiology. 2019;61:225‐227. 30565056 10.1007/s00234-018-2148-4

[32] Graadt van Roggen JF , Hogendoorn PC , Fletcher CD . Myxoid tumours of soft tissue. Histopathology. 1999;35:291‐312. 10564384 10.1046/j.1365-2559.1999.00835.x

[33] Baheti AD , Tirumani SH , Rosenthal MH , et al. Myxoid soft‐tissue neoplasms: comprehensive update of the taxonomy and MRI features. AJR Am J Roentgenol. 2015;204:374‐385. 25615761 10.2214/AJR.14.12888

[34] Parmar HA , Hawkins C , Ozelame R , Chuang S , Rutka J , Blaser S . Fluid‐attenuated inversion recovery ring sign as a marker of dysembryoplastic neuroepithelial tumors. J Comput Assist Tomogr. 2007;31:348‐353. 17538277 10.1097/01.rct.0000243453.33610.9d

[35] Qaddoumi I , Orisme W , Wen J , et al. Genetic alterations in uncommon low‐grade neuroepithelial tumors: BRAF, FGFR1, and MYB mutations occur at high frequency and align with morphology. Acta Neuropathol. 2016;131:833‐845. 26810070 10.1007/s00401-016-1539-z PMC4866893

[36] Daumas‐Duport C . Dysembryoplastic neuroepithelial tumours. Brain Pathol. 1993;3:283‐295. 8293188 10.1111/j.1750-3639.1993.tb00755.x

